# Supplementary figures and images for: Computational epitope mapping of class I fusion proteins using low complexity supervised learning methods
Source: PLoS Comput Biol. 2022 Dec 7;18(12):e1010230. doi: 10.1371/journal.pcbi.1010230 (PMC9762601; doi:10.1371/journal.pcbi.1010230)

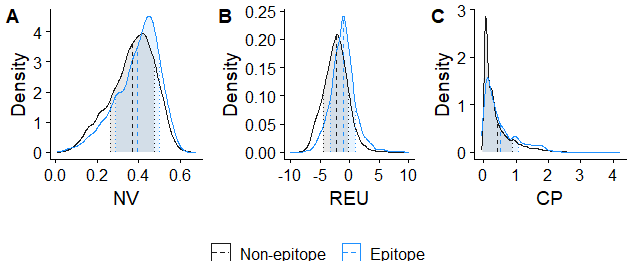

Supplement: S1 Fig — Mean feature values of epitope and non-epitope residues are indicated by vertical dashed lines. Score values within one standard deviation of each group’s mean are shaded and encased by vertical dotted lines. The distribution-free overlapping index η and the Welch’s two-tailed t test p value are indicated in each panel for the distribution overlap and significance of mean difference between epitope and non-epitope residues. For reference, two distributions would be identical if η = 1.00, and unique if η = 0.00. A p value of less than 0.05 indicates significant differences in mean values. A) Neighbor Vector (NV) distributions. B) Per-residue Rosetta Relative Energy Unit (REU) distributions. C) Contact proximity variation (CP) distributions. (PNG) [file pcbi.1010230.s001.png]

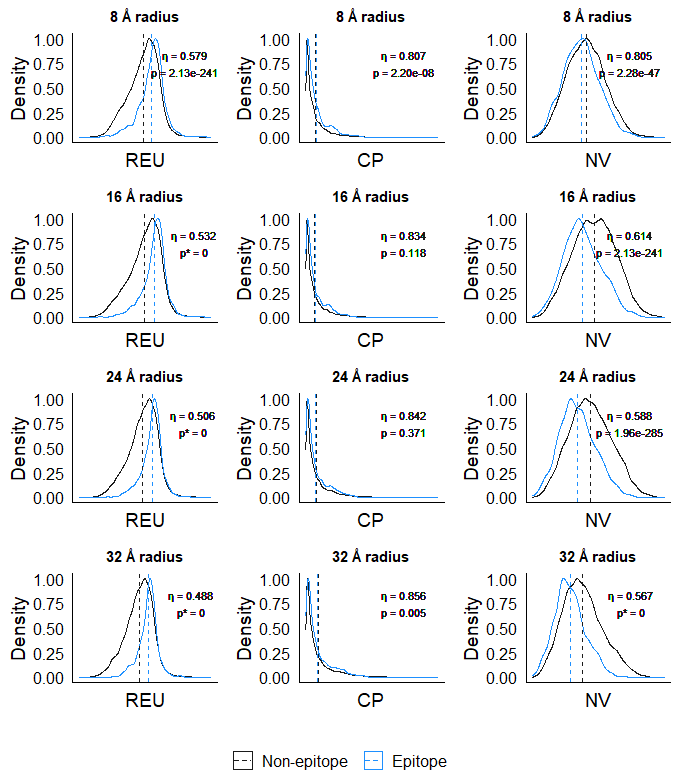

Supplement: S2 Fig — The upper boundary radius used to calculate each NS feature is indicated in each panel’s title. The distribution-free overlapping index η and the Welch’s two-tailed t test p value are indicated in each panel for the distribution overlap and significance of mean difference between epitope and non-epitope residues’ NS values. Vertical dashed lines indicate each distribution’s mean value. For values p*, the p value could not be calculated due to values being too close to zero. (PNG) [file pcbi.1010230.s002.png]

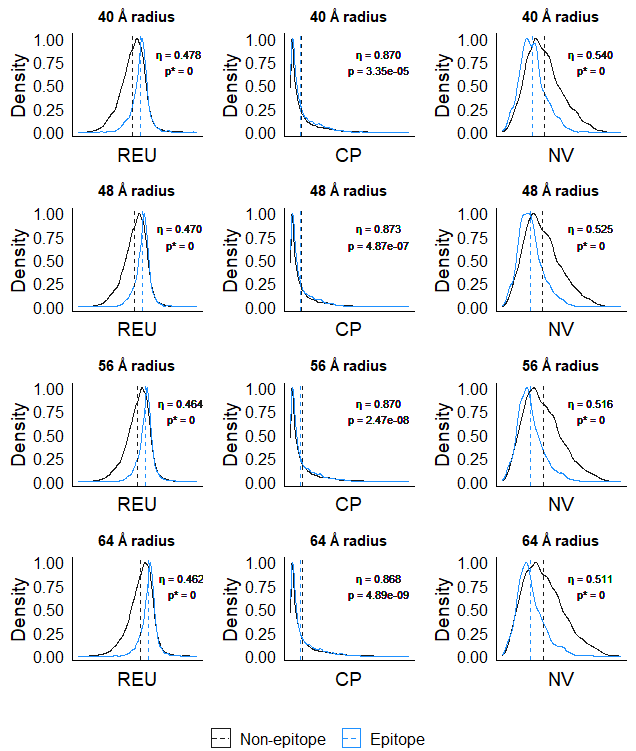

Supplement: S3 Fig — (PNG) [file pcbi.1010230.s003.png]

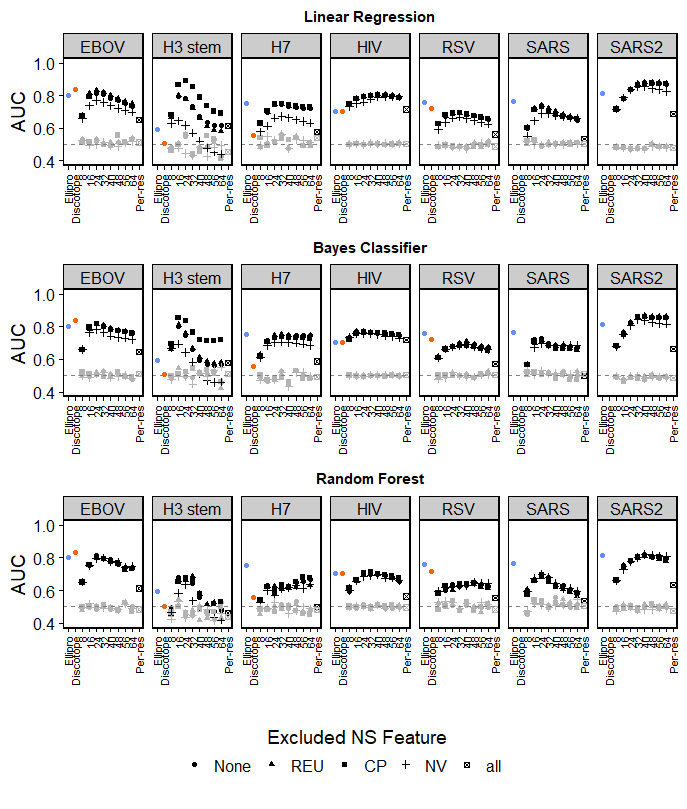

Supplement: S4 Fig — (PNG) [file pcbi.1010230.s004.png]

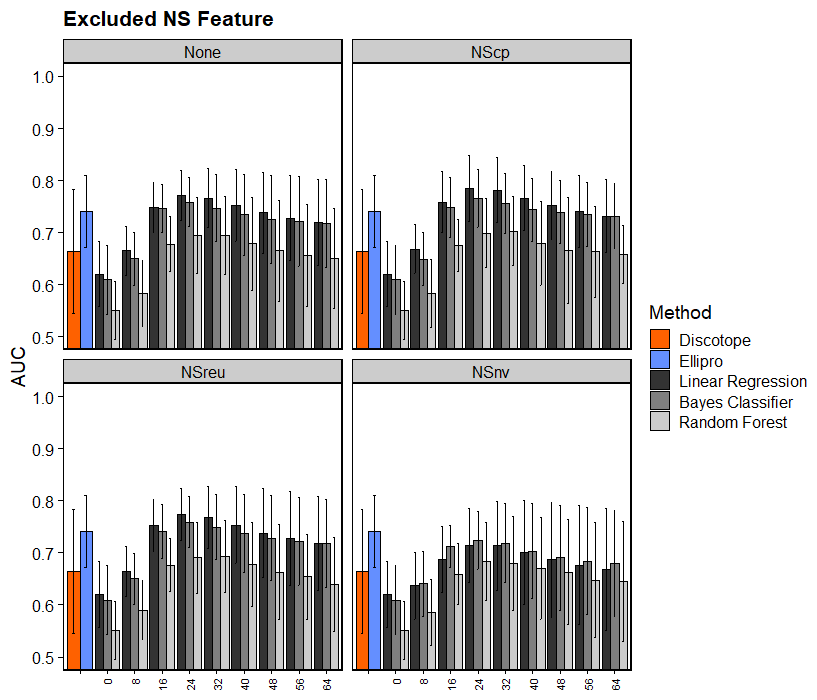

Supplement: S5 Fig — (PNG) [file pcbi.1010230.s005.png]

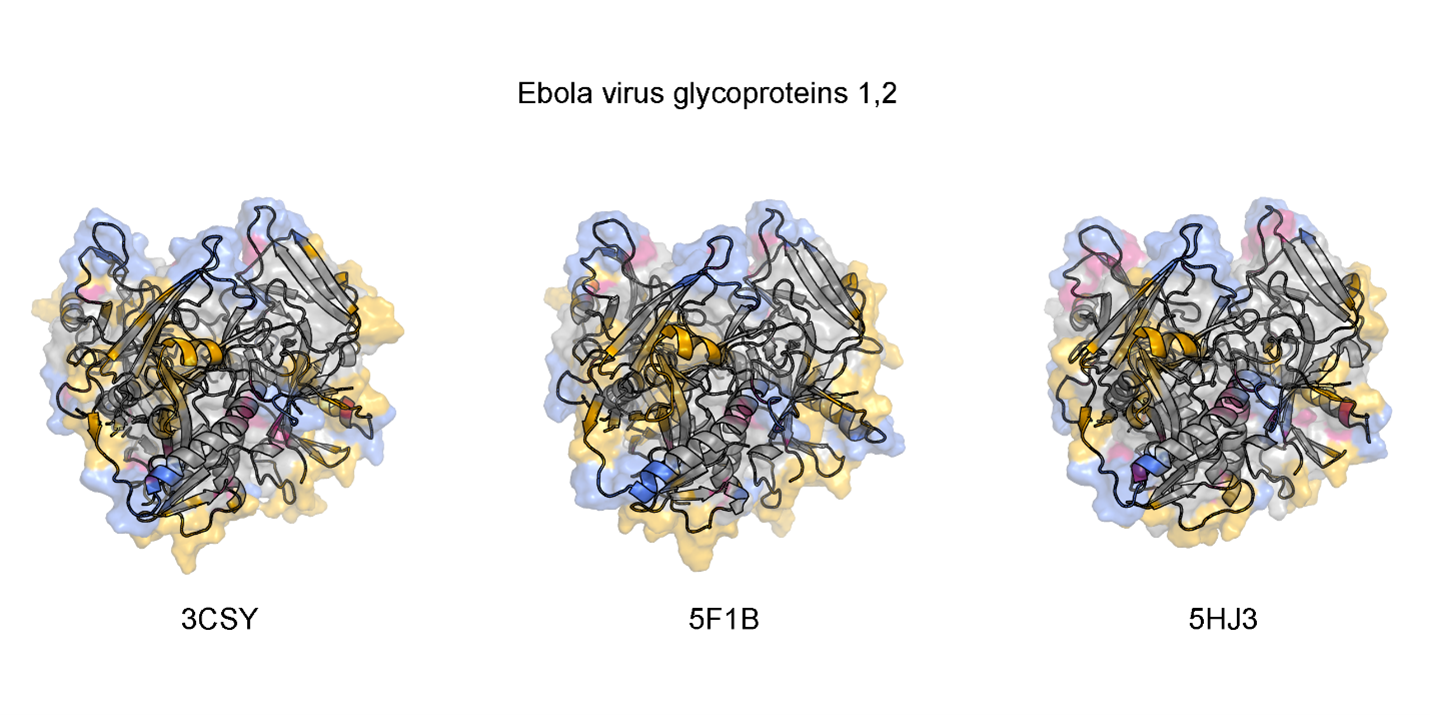

Supplement: S6 Fig — Proteins are oriented so that residues closest the viral membrane are at the bottom. Predictions are color coded using the same scheme as in Fig 3A, with TP as blue, FP as yellow, FN as pink, and TN as grey. (PNG) [file pcbi.1010230.s006.png]

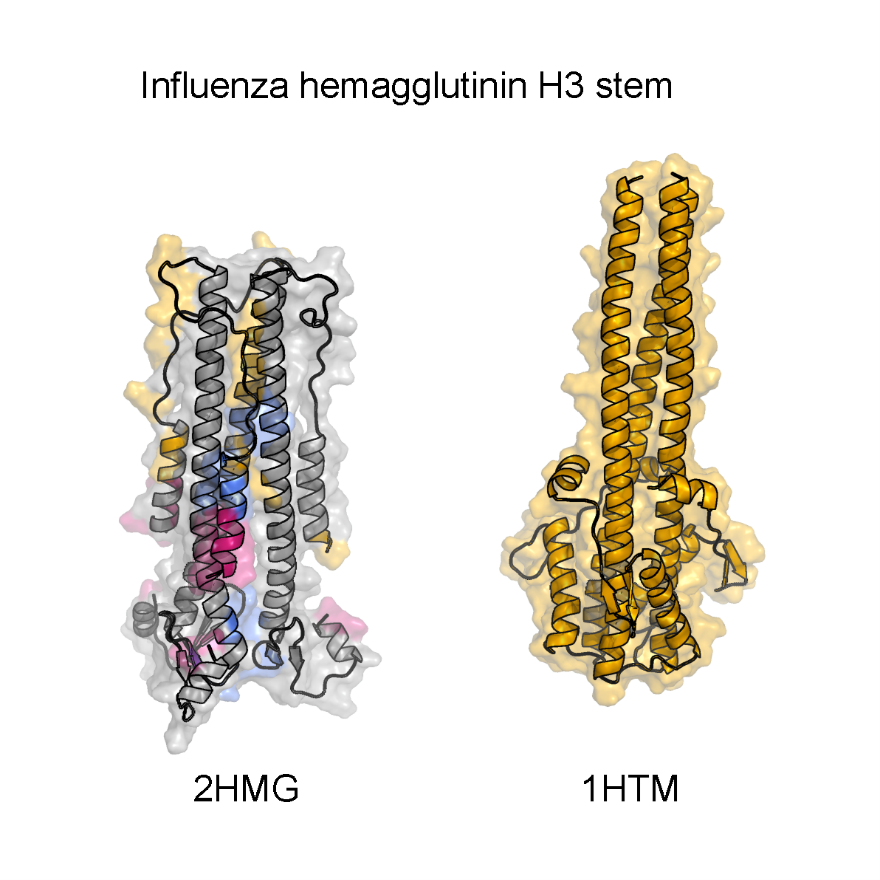

Supplement: S7 Fig — Proteins are oriented so that residues closest the viral membrane are at the bottom. Predictions are color coded using the same scheme as in Fig 3A, with TP as blue, FP as yellow, FN as pink, and TN as grey. (PNG) [file pcbi.1010230.s007.png]

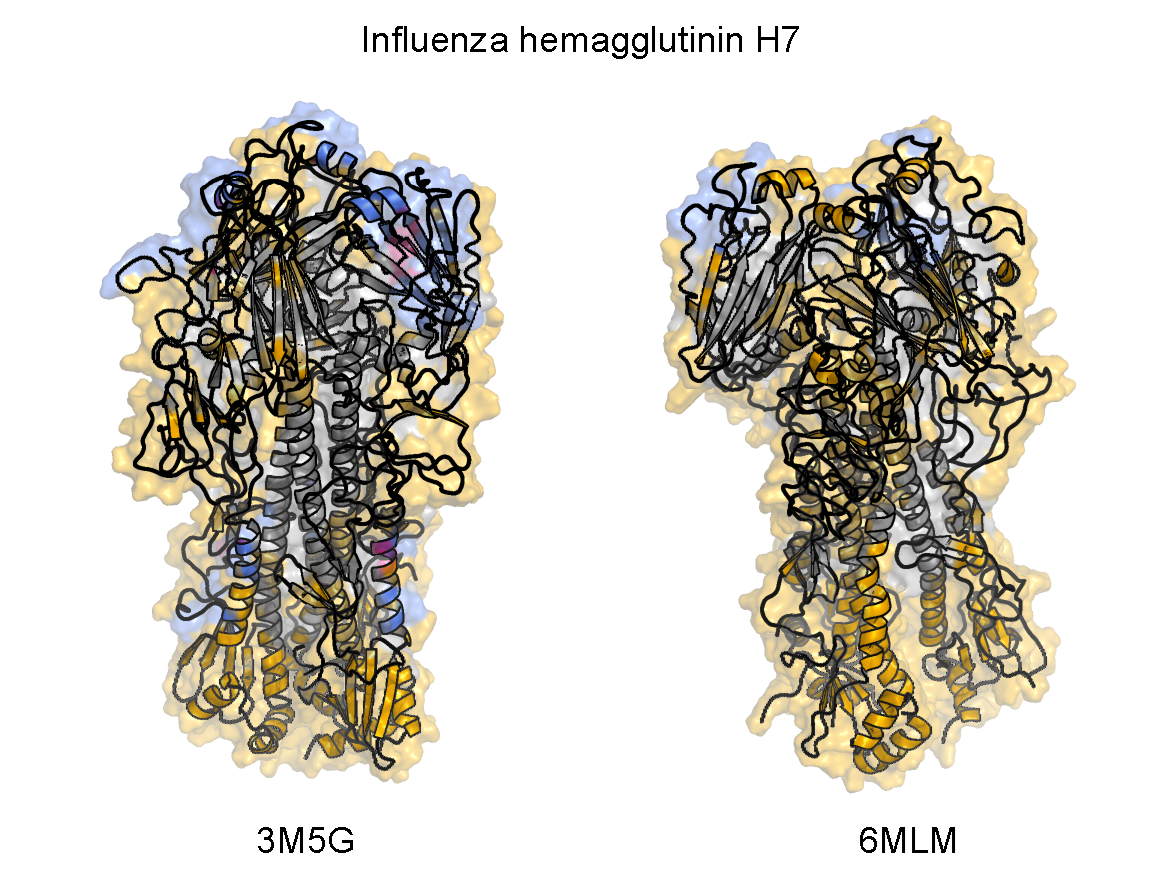

Supplement: S8 Fig — Proteins are oriented so that residues closest the viral membrane are at the bottom. Predictions are color coded using the same scheme as in Fig 3A, with TP as blue, FP as yellow, FN as pink, and TN as grey. (PNG) [file pcbi.1010230.s008.png]

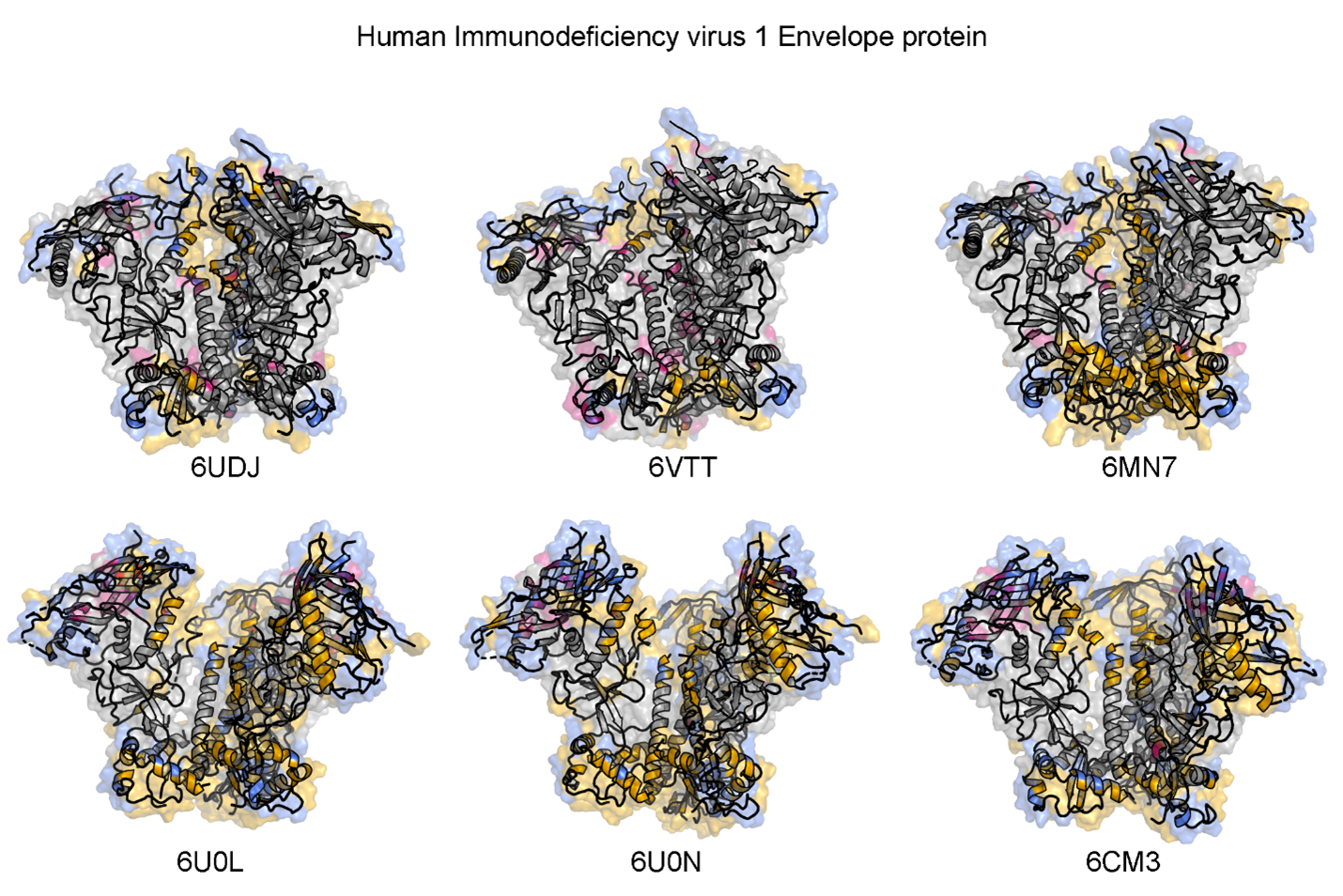

Supplement: S9 Fig — Proteins are oriented so that residues closest the viral membrane are at the bottom. Predictions are color coded using the same scheme as in Fig 3A, with TP as blue, FP as yellow, FN as pink, and TN as grey. (PNG) [file pcbi.1010230.s009.png]

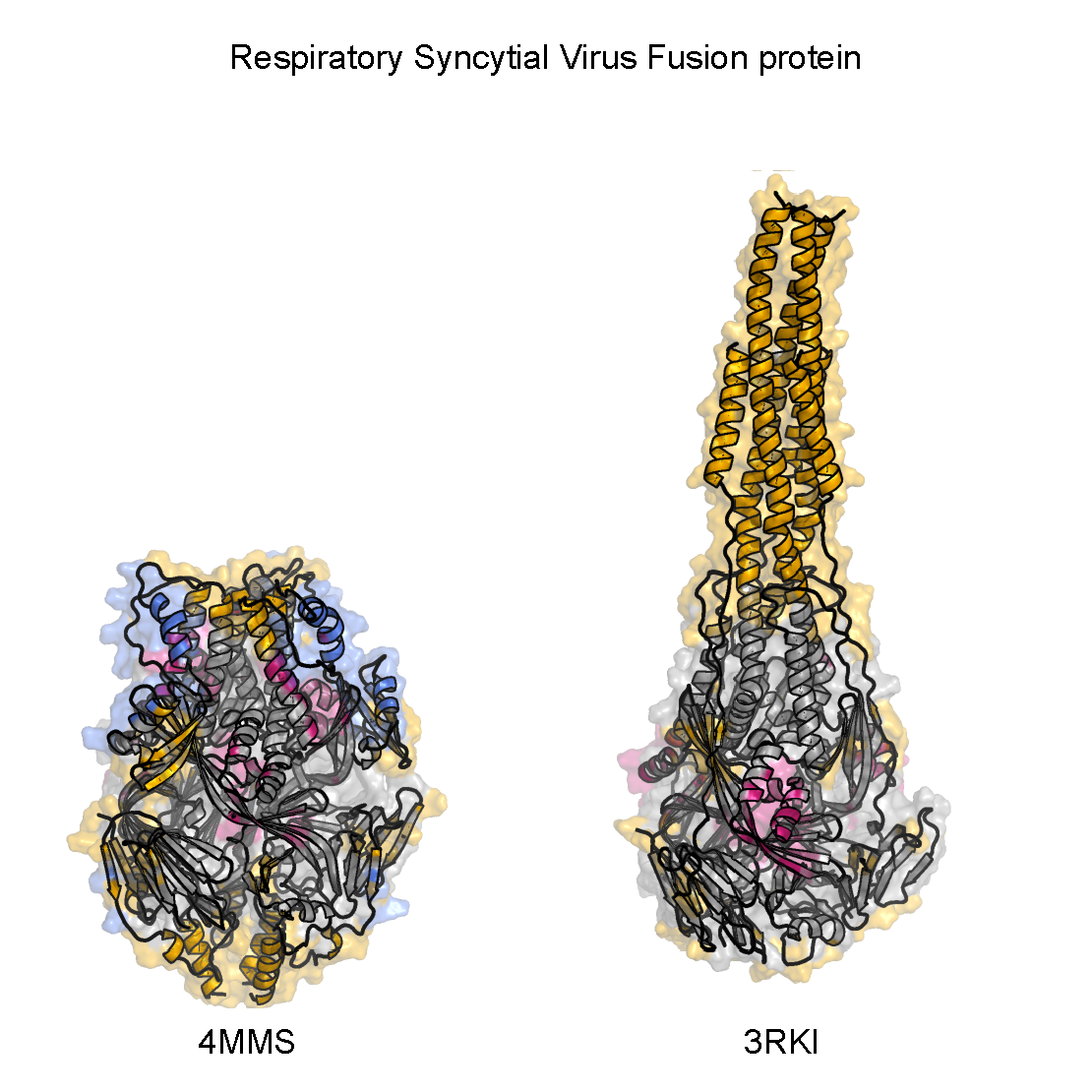

Supplement: S10 Fig — The RSV F structure 4MMS is oriented so that residues closest the viral prefusion membrane are at the bottom. The RSV F structure 3RKI is oriented so that residues closest the viral postfusion membrane are at the top. Predictions are color coded using the same scheme as in Fig 3A, with TP as blue, FP as yellow, FN as pink, and TN as grey. (PNG) [file pcbi.1010230.s010.png]

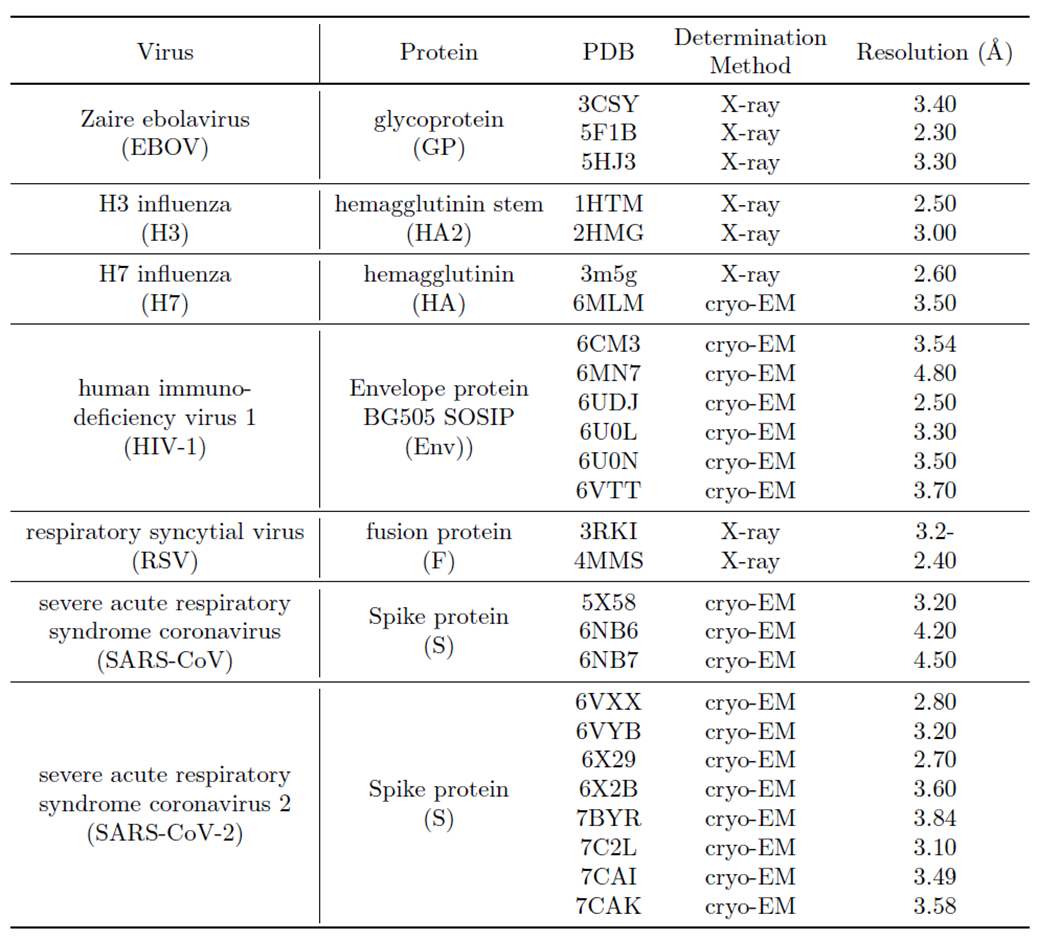

Supplement: S1 Table — (TIF) [file pcbi.1010230.s011.tif]

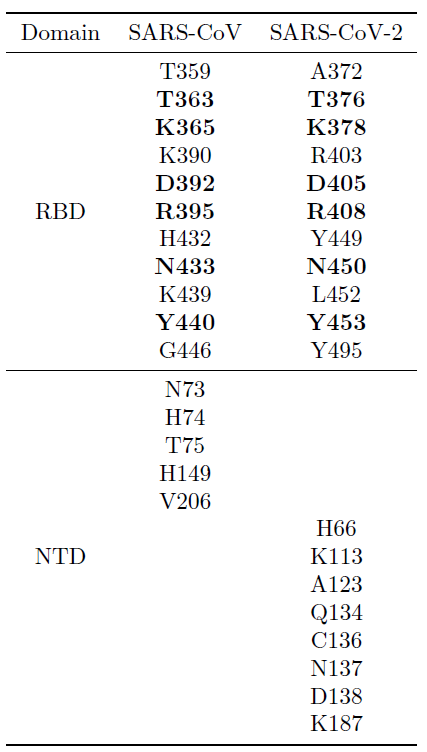

Supplement: S2 Table — Clustal Omega was used to perform a multiple sequence alignment of SARS-CoV and SARS-CoV-2 Spike protein sequences. Aligned residues are indicated when residue identities are present in both the SARS-CoV and SARS-CoV-2 columns. Residues of identical sequence identity are indicated in bold. Residue numbers correspond to PDB ID 6NB7 (SARS-CoV S, 2-up conformation) and 7CAK (SARS-CoV-2, 3-up conformation), and chain B for both models. One-letter sequence identities correspond to the consensus sequence. Note, consensus sequence residue H432 of SARS-CoV S protein was altered from the original 6NB7 sequence Y432, which would be identical to aligned Y449 of SARS-CoV-2. (TIF) [file pcbi.1010230.s012.tif]
