# Supplementary material for: Computational epitope mapping of class I fusion proteins using low complexity supervised learning methods
Source: PLoS Comput Biol. 2022 Dec 7;18(12):e1010230. doi: 10.1371/journal.pcbi.1010230 (PMC9762601; doi:10.1371/journal.pcbi.1010230)
Supplement: S1 Protocol Capture — (PDF) [file pcbi.1010230.s013.pdf]

Protocol capture

1 Preparation of Input PDB files for Feature Calculations

1.1 Initial Curation of aligned PDB files

Class I fusion proteins are trimeric, which may or may not share C3 symmetry, and undergo multiple Ångstrom structural rearrangements during cellular entry. The features used to build the AxIEM model require that all conformations, *i.e.* For all methods described below, all PDB structures used to represent a class I fusion protein during cellular entry must share the same chain and residue order with respect to the superimposition of all structures of the same fusion protein. To meet this criteria, each viral fusion protein used in this study must have at least two PDB structures within the Protein Data Bank that share no less than 1.0 Å RMSD to any other determined structure, and that the PDBs used for the dataset share congruous chain and sequence order with respect to the superimposed PDB ensemble.

Curation of structures began with a PDB search of known class I fusion proteins in their trimeric state, narrowed down by *i)* virus family, *ii)* strain or serotype with at least two structures, and *iii)* all structures share ≥ 95% amino acid sequence identity. Afterwards, all PDB candidates were downloaded from the Protein Data Bank, and aligned using PyMOL using the `align PDB1, PDB2, cycles=0` command for each PDB (PDB1) to all other identified PDBs (PDB2). For PDB structures sharing less than 1.0 Å RMSD, the structure with the least number of missing densities was used. In the case if any two structures shared less than 1.0 Å RMSD and the same number of residues without missing densities, the structure with the lowest resolution was given preference. As a side note, for the majority of class I fusion protein structures available within the PDB, the preponderance of determined structures were of a single domain, most often as a monomeric domain, which eliminated most viral fusion protein candidates from the final dataset used to train and analyze the AxIEM model(s).

Superimposition sometimes required reordering chain identification to preserve residue ordering. Table 1 represents the PDB files originally downloaded from the Protein Data Bank, the original chain order, and the new chain order. If chain reordering was necessary, the following script was used to reorder the chain IDs.
The notation '\' indicates that the following line should be entered on the same line when entered in the terminal.

```
python reorder_pdb_chains.py <input.pdb> <output.pdb> \
--new_chain_order=NEW_CHAIN_ORDER \
--new_chain_id=NEW_CHAIN_ID --preserve
```

After reordering the chains, the sequence of each conformation's monomer (including either the protomer FASTA file or the two cleaved attachment and fusion domain chains concatenated together as one FASTA file) was aligned using Clustal Omega. All residues which aligned were considered for epitope prediction. Any residues that were not present in all PDB structures were removed from the respective PDB structure.

1.2 Identification and threading of consensus sequence onto native models

Given that some of the conformations were engineered to be conformationally stable, the original sequences of the experimentally determined PDB ensemble did not share 100% sequence identity. Therefore, the consensus sequence of full-length protein isolated from human hosts was determined and threaded onto each native model backbone, that is, the consensus sequence was used to replace the original PDB sequence so that the amino acid identity of models were identical. In all cases the consensus sequence shared ≥ 98% sequence identity.

Database	Date Accessed	Virus (taxid)	Host (taxid)	Collection Dates	# Full-length Sequences
NCBI Virus	07/13/2020	SARS-CoV-1 (694009)	Human (9605)	01/01/2000 - 01/01/2019	9
NCBI Virus	07/13/2020	SARS-CoV-2 (2697049)	Human (9605)	all	9,339
NCBI Virus	09/10/2020	Human RSV A (208893)	Human (9605)	all	767
NCBI Virus	09/10/2020	HIV-1 (11676)	Human (9605)	all	80760
NCBI Virus	09/10/2020	Zaire EBOV (186538)	Human (9605)	all	1700
Influenza Virus Resource	09/10/2020	Influenza A H3N*	Human	all	28519
Influenza Virus Resource	09/10/2020	Influenza A H7N*	Human	all	108

Table 1: Accession Dates of Virus Sequences. Default parameters were used unless noted above to query for sequences of each viral fusion protein.

To generate the consensus sequence, a multiple sequence alignment was performed using a locally-installed version of Clustal Omega (`http://www.clustal.org`) to align all full-length sequences obtained from the NCBI Virus, or other specialized NCBI-sponsered database (Table 1). Sequences were were initially downloaded as a Protein FASTA file and then aligned using the following command.

```
clustalo -i <sequence.fa> -o <sequence.aln> -t Protein --infmt=fa
```

Next, the consensus sequence of each multiple alignment was obtained using `EMBOSS v.6.6.0.0` with the `cons` package (`ftp://emboss.open-bio.org/pub/EMBOSS/`).

```
cons -sequence <sequence.aln> -outseq <sequence.cons>
```

To thread the consensus sequence over each PDB, the consensus sequence was first aligned to each of the native PDB sequences. This required that the consensus sequence was concatenated in triplicate since the consensus sequence only represented the full-length sequence of a viral fusion protein monomer, while the PDB sequence represented a trimeric sequence. Each PDB FASTA files was obtained using the following command:

```
python get_fasta_from_pdb.py <pdb>
```

The consensus and full-length native sequences were aligned using Clustal Omega (`<virus>_cons.fasta` and `<virus>_cons.aln`). Afterwards, a `grishin` file was created using the consensus sequence as the target sequence for all native templates, and the Rosetta Partial Thread application was used to assign coordinates to the consensus sequence. For an alternative protocol capture on partial threading, see Section 2 of this tutorial. The output PDB model was used to obtain all features, except for the Rosetta REU residue score that requires minimization for scoring (as described in the following section), and contains the renumbered sequences as listed in Table 2.

```
/path/to/rosetta/main/source/bin/partial_thread.linuxgccrelease \
-database /path/to/rosetta/main/database -in:file:fasta <sequence.cons> \
-in:file:alignment cons_<pdb>.grishin -in:file:template_pdb <pdb>
```

Viral protein	Chain	Residues considered for design
EBOV Zaire GP	A	32-188, 516-521, 525-597
	B	708-864, 1192-1197, 1201-1273
	C	1384-1540, 1868-1873, 1877-1949
influenza A H3 HA2	A	387-499
	B	958-1070
	C	1529-1641
influenza A H7 HA	A	19-229, 238-334, 354-519
	B	587-797, 806-902, 922-1087
	C	1155-1365, 1374-1470, 1490-1655
HIV-1 Env	A	34-57, 68-78, 82-127, 192-300, 325-353,356-394, 412-457, 464-501
	B	35-57, 68-78, 82-127, 194-300, 325-394,412-457, 464-501
	C	35-57, 68-78, 82-126, 194-301, 325-394,412-457, 464-501
RSV F	A	27-97, 155-322, 333-505
	B	601-671, 729-896, 907-1079
	C	1175-1245, 1303-1470, 1481-1653
SARS-CoV-1 S	A	30-137, 149-167, 179-236, 250-482 491-502, 509-662, 673-808, 835-1104
	B	1285-1392, 1404-1422, 1434-1491, 1505-1737 1746-1757, 1764-1917, 1928-2063, 2090-2359
	C	2540-2647, 2659-2677, 2689-2746, 2760-2992 3001-3012, 3019-3172, 3183-3318, 3345-3614
SARS-CoV-2 S	A	27-66, 82-95, 99-113, 116-140, 167-172, 187-196, 200-209, 217-242, 264-328, 335-441, 449-454, 491-498, 503-515, 522-620, 641-672, 690-810, 814-827, 856-1146
	B	1300-1339, 1355-1368, 1372-1386, 1389-1413, 1440-1445, 1460-1469, 1473-1482, 1490-1515, 1537-1601, 1608-1714, 1722-1727, 1764-1771, 1776-1788, 1795-1893, 1914-1945, 1963-2083, 2087-2100, 2129-2419
	C	2573-2612, 2628-2641, 2645-2659, 2662-2686, 2713-2718, 2733-2742, 2746-2755, 2763-2788, 2810-2874, 2881-2987, 2988, 2995-3000, 3037-3044, 3049-3061, 3068-3166, 3187-3218, 3236-3356, 3360-3373, 3402-3692

Table 2: Residue positions considered for design. All PDB models within an ensemble are numbered identically, and all chain identifiers from the initial model are eliminated. Chain identification denote individual monomers of Class I fusion proteins. Residue numbering is based off of the threaded model (*i.e.* the `<pdb>_threaded.pdb` model), or rather a residue's position in the full-length consensus sequence. For Class I fusion proteins, only residues that are present in all three protomers were considered for design. Residues not present within all protomers were kept in the native model were allowed to repack (re-position) their side chains during design. HIV-1 Env was not subjected to threading due to low consensus sequence identity, and the numbering of the models used retained their original PDB numbering.

1.3 Energy minimization and scoring to obtain the Rosetta REU residue score

Note, this section requires the use of the Rosetta protein structure prediction and design modeling suite, which is available by license for free for non-commercial purposes, although a commercial license is available. If you are new to using Rosetta or would like to learn more about how install and use Rosetta, please start here. For the following code blocks, you will need to replace `/path/to/` with the correct directory path to where you have installed Rosetta on your own machine. Depending on the build you installed, you will need to replace `linuxgccrelease` with the release version you installed. The threaded template models were subjected to constrained Rosetta FastRelax to generate 50 relaxed models.

```
/path/to/rosetta/main/source/bin/rosetta_scripts.default.linuxgccrelease \
@relax.flags -s <threaded pdb> -scorefile <pdb>_relaxed.fasc

---------------relax.flags---------------
 -database /path/to/rosetta/main/database/
 -linmem_ig 10
 -in:file:fullatom
 -in:detect_disulf false
 -relax:fast
 -relax:constrain_relax_to_start_coords
 -out:file:fullatom
 -out:suffix _relax
 -use_input_sc
 -nstruct 50
----------------------------------------
```

The relaxed model with the combined lowest total energy score and lowest C_α root mean square deviation (RMSD) to the threaded PDB structure were selected as the input model to calculate per-residue REU as follows:

```
python /path/to/rosetta/tools/protein_tools/scripts/score_vs_rmsd.py \
-n <threaded_pdb> -c ca -t total -o <pdb>_sc_rmsd.tab <pdb>_threaded_relax_*pdb

cat <pdb>_sc_rmsd.tab| tail -50 | sort -k2 -k3 | head -1 > low_model.txt
cat low_model.txt | awk '{system("cp "$1" <pdb>_relaxed.pdb")}'
rm low_model.txt
```

The per-residue energy scores were obtained using the per-residue total energies of the Rosetta `score_jd2` output score file, (`<pdb>_relaxed.sc`):

```
/path/to/rosetta/main/source/bin/score_jd2.linuxgccrelease \
-s <pdb>_relaxed.pdb -ignore_unrecognized_res \
-out:file:scorefile <pdb>_relaxed.sc
```

2 Assignment of conformation dependent epitope residues

An epitope reside is first defined here as any residue that has been annotated as an epitope by the Immune Epitope DataBase (IEDB), Influenza Research Database's Immune epitope search, or the HIV Molecular Immunology Database that is associated with a PDB structure. (Table 3). IEDB searches used the filters 'Positive Assays only', 'Epitope Structure: Discontinuous', 'No T cell assays', 'No MHC ligand assays', and 'Host: Homo sapiens (human)'. Influenza epitope searches used the filters 'Virus Type A', 'Subtype H3 or H7', 'Protein HA, Segment 4', 'Experimentally Determined Epitopes', 'Assay Type Category and Result B-cell Positive', and 'Host Human'. HIV epitopes include epitopes as listed in the interactive epitope maps as of 1 June, 2020.

To determine each epitope residue's conformation specificity, a residue must have at least one PDB structure of an antibody-antigen complex where it has been annotated as an epitope residue (*i.e.* it has an IEDB ID or is listed as an epitope

on the HIV DB gp160's epitope interaction map), and that the PDB of the annotated antibody-antigen complex, when aligned to each monomer or chain of the benchmark protein models, results in zero overlap of the antibody with each benchmark protein model. Checking for overlap was performed as follows. *i*) For each PDB antibody-antigen complex associated with an IEDB ID or HIV epitope, the antigen and the antibody were created as independent PyMOL objects, let's say labeled as objects `antigen` and `antibody`. *ii*) Three PyMOL objects were created for each AxIEM benchmark PDB of a viral fusion protein, with each object containing the residues identified to be present in the antigen of the antibody-antigen complex, labeled as `objA`, `objB`, and `objC`, respectively. *iii*) The `antigen` object was first aligned to each `objX` object. *iv*) Next, the `antibody` object was aligned to `antigen` with respect to it's aligned position to `objX`. *v*) If no atoms of the `antibody` object came within 3 Å of any atoms present in the AxIEM PDB model, the residues within `objX` were considered to be a viable conformation-dependent epitope. There were often multiple (subunit) antibody-antigen complexes associated with each IEDB ID, and if any one of the representative complexes met the criteria in step *v*, those residues for the given monomer/protomer were assigned as epitope residues due to the potential of differing antibody binding angle.

Viral Protein	Epitope ID	Epitope Residues	Excluded conformations
Zaire EBOV GP	442029	N550, D552, G553, C556	
	534853	A526, I527	
	534854	K114, K115, P116, D117, G118, E120, S142, G143, T144, G145	
	534855	H549, N550, Q551, D552, G553, L554, I555, C556	
	539006	N550, D552	
	606556	G528	
	857622	N550, D552, G553, C556	
	933255	A148, G149, I532	
	933256	G118, T144	
	933257	G149, I532	
	933258	A525, I527, I532	
	933259	I185, I527, I532	
	933260	K115, D117, G118, T144	
	933263	R64, I527, I532	
	933264	S46, D49, G118, T144	
	985426	K114, P116, D117, G118, S119, T144, P146, A148	
	985702	P116, D117	
	1063108	A525, A526, I527, G528, L529, A530, W531	
influenza H3 HA2	189321	T387, Q388, I391, D392, I394, N395, G396, L398, N399, I402, K404, T405, N406, R499	1HTM
	580002	Q388, I391, I394	1HTM
	580003	T387, Q388, I391, D392, I394, N395, G396, L398, N399, I402, E403, K404	1HTM
	742477	I391	1HTM
influenza H7 HA	H7.5	S136, G137, N164, N167, A169, F170, P171, Q172, M173	3M5G
	580003*	D366, G367, W368, D384, T388, Q389, I392, G397, L399, N400, I403, K405	6MLM
	886618	A143, T144, S145, R148, S152, W160, L162, T165, D166, A168, A198, E199, K202, L203	6MLM

	886619	I138, R139, N141, G142, A143, T144, S145, S152, W160, N164, T165, D166, N167, A168, S196, T197, A198, E199, T201, K202	6MLM
influenza H7 HA	886620	R139, N141, G142, A143, T144, S145, R148, S150, G151, S152, W160, L162, D166, N167, V195, S196, A198, K202, L203	6MLM
	952484	G151	6MLM
	164069	C119, V120, L122, M434, P437	6U0L, 6U0N
	489886	E87, N88, T90, P238, P240	
	164067	C119, V120, L122, T198, 199, A200, 201	6U0L, 6U0N
	16470	C119, V120, T198, 199, A200, 201, 202, M434, A436, P437, P438	6U0L, 6U0N
	164071	I109, W112, V255, A281, S365, G366, G367, D368, E370, V/I371, G382, Y384, K421, N425, G473, D474, M475, R476	6U0L, 6U0N
HIV-1 Env	164073	T283, S365, G366, G367, D368, L369, E370, V/I371, T372, T373, Y384, R419, K421, N425, M426, G473, N474	
	164094	V120, L122, P124, L369, I420, I423, I424, N425, I430, M434	6U0L, 6U0N
	164099	K97, T123, N276, T278, N279, N280, A281, K282, S365, G366, G367, D368, W427, W428	6U0L, 6U0N
	227937	V44, W45, R46, E47, V89, T90, E91, N92, F93, N94, K97, N234, T236, G237, P238	6U0L, 6U0N
	534824	Q82, E83, I84, V245, Q246	6U0L, 6U0N
	489875	D325, I326, R327, Q328, H330, T413, P415	
	186804	N63, K65, E66, K68, K196, N197, Y198, I199, D200, K201, Q202, L203, L204, P205, I206, V207, N208, K209, Q210	3RKI
	77299	I266	
	429158	T50, L305, G307, I309, D310	
	566539	K271, L467, K470	3RKI
	566540	L258, I261, N262, D263, P265, I266, T267, N268, D269, K271, K272, S275	4MMS
	581507	N175, D263	
	581508	S169, A170, L172, S173, T174, N175, K176, A177, V178, L188, K191, D194 N197, K201, K226, D263	3RKI
RSV F	581509	S173, T174,	
	581510	S173, T174, N175	
	581511	T174, D263	
	581512	T174, N175, D194, D263	
	591404	G307, D310	
	606552	N63, E64, K65, E66, K68, C69, N197, D200, K201, Q202, L204, P205, I206, N208, K209, Q210, C212, E295	3RKI
	912903	L61, S62, N63, I64, K65, E66, L83, D84, Y86, K87, V90, Q94, E161, G162, N165, K168, S169, L172, L204, N208, I292, K293, E294, E295	3RKI

RSV F	969092	N63, K65, K196, D200, K201, L204, P205, N208, K209, Q210, E294, E295	3RKI
	753466	E31, Y33, S35, K42, P312, W314, D344, S377, E378, N380, L381, N383, V384, K390	4MMS
SARS-CoV S	76972	D462	6NB7, 6NB6(B,C)
	77442	R426, S432, T433, Y436, N437, K439, Y440, Y442, P469, P470, A471, L472, N473, C474, Y475, W476, L478, N479, D480, Y481, G482, Q492	6NB7, 6NB6(B,C)
	77444	T359, T363, K365, K390, G391, D392, R395, R426, Y436, G482	6NB7, 6NB6(B,C)
	420672	K344, F360, Y442, L472, D480	6NB7, 6NB6(B,C)
	420673	N479	6NB7, 6NB6(B,C)
	910052	G446, P462, D463, Y475	6NB7, 6NB6(B,C)
	1074318	D480	6NB7, 6NB6(B,C)
	1074319	K439, G446, S461, D463	6NB7, 6NB6(B,C)
SARS-CoV-2 S	997006	Y369, N370, S371, A372, F374, F377, K378, C379, Y380, G381, V382, S383, P384, T385, K386, L390, F429, T430, F515	all but 7CAI(B), 7CAK
	1074327	Y369, N370, A372, F374, T376, F377, K378, Y380, V382, P384, T385, K386, D389, L390, F392, D428, F429, T430, F515	all but 7CAI(B), 7CAK
	1075135	R346, Y449, N450, L452, S494	6VXX, 6VYB(A,C), 6X2B(A), 7C2L(C)
	1075136	R403, Q409, T415, G416, K417, D420, Y421, Q493, Y495, G496, Q498, Y505	6X2B(A), 6X29, 7BYR(B,C), 7C2L((B,C), 7CAI(C)
	1083498	R403, D405, E406, R408, Q409, T415, G416, K417, D420, Y421, L455, Q493, Y495,Y505	6VXX, 6VYB(A,C), 6X2B(A), 6X29, 7BYR(B,C), 7C2L(B,C), 7CAI(C)
	1087140	Y449, L492, Q493, S494, G496, Q498, Y505	6VYB(A,C), 6X2B(A), 7BYR(A,C), 7C2L(C), 7CAI(C)
	1097186	Y369, N370, S375, T376, F377, K378, C379, Y380, G381, V382, S383, P384, T385, K386, L390, F392, D428, T430	all but 7CAI(B)
	1087266	R403, D405, R408, T415, G416, K417, D420, Y421, Y453, QQ493, S494, Y495, G496, Q498, Y505	6VXX, 6VYB(A,C), 6X2B(A), 6X29, 7BYR(B,C), 7C2L(A), 7CAI(A,B)
	1087267	R403, D405, T415, G416, K417, D420, Y421, Y453, Y495, Y505	6VYB(B), 6X2B(B,C), 7BYR(A), 7C2L(A), 7CAI(A,B)
	1087269	Y369, N370, F374, S375, T376, F377, K378, C379, Y380, G381, V382, S383, P384, T385, K386, L390, F392, D428, T430	all but 7CAI(B), 7CAK
	1087820	D428, F429	6VXX
	1087821	N354	6VXX
	1125015	A372, F374, C379	6vXX
	1125016	F374, S375, T376, F377, C379, F392, D427	6VXX

SARS-CoV-2	1181325	Y449, Y453, L492, Q493, S494	6VYB, 6X2B(A,C), 7BYR(A,C), 7C2L(A,C), 7CAI, 7CAK
	1307796	Y369, S375, F377, K378, C379, Y380, G381, V382, S383, P384, T385, K386, F392, P412, G413, D427, D428, F429	all but 7CAI(A,B)
	1309150	Y369, N370, F374, S375, T376, F377, K378, C379, Y389, G381, V382, S383, P384, T385, K386, L390, R408, D428, T430	6VXX, 6VYB, 6X29, 6X2B
	1310037	Y449, Q493	6VYB(A,C), 6X2B(A), 7BYR(C), 7C2L(C), 7CAI(C)
	1310038	R403, D405, T415, G416, K417, D420, Y421, Y453, Q493, S494, Y495, G496, T500, Y505	6VXX, 6VYB, 6X29, 6X2B(A), 7BYR(B,C), 7C2L(A,B), 7CAI(C)

Table 3: Residues classified as epitopes. All residues listed were annotated to be experimentally determined epitope contacts that are present in all PDB models used for the AxIEM benchmark. Residue numbering refers only to Chain A or the original consensus sequence position number. The column 'Excluded Conformations' refers to any conformations (PDB ID) for which those residues did not meet the criteria to be classified as an epitope. The `AxIEM.data` file contains each residue's epitope label, or classifier, with a `1` indicating that residue was assigned to be a conformation-specific epitope residue or `0` if not. With the exception of HIV-1 Env, all proteins use contiguous numbering —instead of restarting the same numbering scheme with each chain —so that the labels had to be mapped to the correct position index within the dataset and models' PDB numbering schema, which is reflected in `AxIEM.data` but not in the table.

3 AxIEM Benchmark

3.1 Benchmark with Discoscope and Ellipro 2.0

The initial annotated dataset `AxIEM.data` was constructed by pasting and concatenating the classifier labels and pre-computed Rosetta per-residue total score energies for all PDB structures, to which virus protein name, PDB ID, and PDB residue IDs labels were added for data clarity. Next, the contact proximity variation and neighbor vector features were calculated using the following script.

```
python src/AxIEM_Step1_benchmark.py --data AxIEM.data --features AxIEM_per-residue.features
```

Afterwards, Neighbor Sums were calculated and appended to generate the complete dataset `AxIEM_updated.features`.

```
python src/AxIEM_Step2_benchmark.py --data AxIEM_per-residue.features
--features AxIEM_updated.features --randomized_features AxIEM_randomized.features \
--plotting feature_distributions.txt
```

Finally, linear regression, Bayes classifier, Logistic regression, and random forest classifier models were trained and test using leave-out tests. Individual leave-out performance tests can be found in the `results/benchmark_all_leaveout_AUCs`.

```
python src/AxIEM_Step3_benchmark.py --data AxIEM_updated.features \
--randomized_data AxIEM_randomized.features --discotope Discotope.data \
--ellipro Ellipro.data --summary benchmark_leaveout_AUCs.txt \
--averages benchmark_avgAUC.txt --rocs benchmark_rocs.txt
```
