# Supplementary material for: Computational epitope mapping of class I fusion proteins using low complexity supervised learning methods
Source: PLoS Comput Biol. 2022 Dec 7;18(12):e1010230. doi: 10.1371/journal.pcbi.1010230 (PMC9762601; doi:10.1371/journal.pcbi.1010230)
Supplement: Attachment — Submitted filename: Letter_to_the_Reviewers_AxIEM.pdf [file pcbi.1010230.s014.pdf]

Dear Reviewers,

Thank you for your insightful comments and the time you provided to create your reviews. We have addressed your comments and concerns as diligently as possible, discussed below.

1) *Usage of Bayes classifier is rightfully motivated by the low sample size. However, how accurate is the multivariate gaussian distribution assumption for this data set? Especially given that the NV feature is 0-1 bounded with an heavy left tail, and that the CP RMSD feature is clearly bimodal for non-epitope residues (Sup. Fig1). Have other supervised ML approaches such as logistic regression or random forest been evaluated here?*
    a. We included additional methods including linear regression, logistic regression, and random forest classification, shown in the updated version of Fig2, S4 Fig, and S5 Fig. Unfortunately, logistic regression would have been an excellent choice as a classifier, but the Scikit learn logistic regression model failed to converge using the updated feature set (with each feature scaled using the MinMaxScaler algorithm) during training.
    b. Regarding the CP RMSD metric, we followed the suggestion from Comment 2 and replaced CP RMSD with CP variation. In doing so, the bi-modal distribution of non-epitope residues is reduced.
    c. Most importantly, due to this comment we would like to propose altering the title of the manuscript to "Computational epitope mapping of class I fusion proteins using low complexity supervised learning methods" to reflect that multiple statistical methods were tested to generate the AxIEM model.
2) *Regarding the third feature. The calculation of the standard deviation in Eqn 4 critically depends on the conformational ensemble selected. For the SARS-CoV-2 spike protein, hundreds of cryo-EM structures are available, raising the question of how the 8 structures were selected. Were all the main conformational states represented (from fully closed to fully open)? If yes, how was the balancing done between the four main states? (0,1,2,3 open RBDs?).*
    a. We have updated the Methods subsection Model Preparation (response 2b) and Results subsection Description of AxIEM Dataset (response 2c) to better describe how each conformational ensemble was selected, and have noted that balancing conformations like the 0,1,2,3 open RBD conformations was included to avoid overrepresentation of any one conformation in the CP variation metric.
    b. Lines 444-454: Curation of viral fusion protein conformations began with a Protein Data Bank query of viral fusion proteins in their trimeric state that were identical in virus family and strain or serotype. All PDB structures that shared ≥ 95% amino acid sequence identify were downloaded from the Protein Data Bank and were superimposed in a pairwise fashion using the PyMOL align command with number of cycles set to zero. For PDB structures sharing less than 1.0 Å RMSD, the structure with the least number of missing densities was selected to represent a single fusion protein conformation. The final set of selected fusion protein ensembles included ensembles with at least a single 2.0 Å pairwise RMSD between two conformations, and no two conformations shared less than 1.0 Å RMSD. Additionally, for ensembles such as SARS-CoV-2 that have

multiple, distinct conformations the number of distinct conformations were equally balanced in conformation representation.

c. Lines 112-128: *Selection of protein structures.* The dataset used to build the AxIEM model includes six structure-based features that were obtained from a total of 34 PDB structures representing eight fusion protein ensembles with 82 unique annotated epitopes (**S1 Table**). The structures were selected based on the following criteria so that each protein ensemble included: i) at least two multimeric conformations of greater than 2.00 Å root mean square deviation (RMSD), ii) no two conformations of less than 1.00 Å RMSD, iii) every PDB had a resolution of below 4.5 Å, and iv) each conformation, such as an 'open' or 'closed' conformation, were equally represented within a protein ensemble (26–46). The first criterion was imposed to maximize the structural diversity of each fusion protein ensemble, given that each protein includes at least one side chain contact rearrangement of over 10 Å between the precursor or prefusion conformation and the postfusion conformation. (47–52) Due to the limited number of fusion proteins that have been determined in both their prefusion and postfusion conformations, the threshold of 2.00 Å RMSD was used to allow for selection of multiple prefusion conformations of a single fusion protein, with a unique conformation defined by the second criterion. The last two criteria were needed since the feature set used to train AxIEM require structures with a resolution suitable for identifying side chain orientation without overrepresenting any single conformation's side chain contact orientations within an individual fusion protein ensemble.

3) L355: "In the case where only two conformations were used, only the mean CP value was calculated." The variance is well-defined even for only two samples; why not use it? Also, for such small number of conformations, it is more reliable to use the unbiased estimate for the variance, by dividing by n-1 rather than n.

a. Thank you for this suggestion. We have replaced the contact proximity RMSD metric with contact proximity variance.

4) 4) Eqn5 L363: Eqn5 (weighted averaging over a neighborhood of the above terms) involves an unweighted sum between two unit-less numbers and an energy term (in REU). It makes no sense physically to add them up, and this may result in suboptimal performance. Weights should be introduced here (e.g. by dividing by the standard deviation over the dataset of each term). Another option is to compute three distinct values (one averaging for each term) and calculate 6 features per residue.

a. Thank you very much for this suggestion, as you were correct that using the unweighted sum resulted in suboptimal performance. We chose to follow your second suggestion to replace the unweighted sum of Neighbor sums as three distinct Neighbor Sum scores so that each model could detect individual weights for each Neighbor Summed feature. In the first submission, we did not mention this point.

b. Regarding measuring cumulative performance of AxIEM in relation to Discotope and Ellipro: in the first submission, we incorrectly reported the cumulative performance of AxIEM in the original ROC curves presented in Fig 2B and 2C. These curves were obtained by appending all individual leave-out test predictions and classifier labels as single lists that were used as the expected outcome and prediction to generate the ROC curve and calculate the AUC scores. In this revision, we report the average AUC scores

for epitope predictions by each method, which more accurately reflects the cumulative performance of each method as reported in the updated Fig 2B.

5) 5) The caption of Sup Fig1, writes: "Per-residue Rosetta Relative Energy Unit (REU) distributions. Since each conformation is associated with a unique relative free energy, we used REU to approximate the relative stability of each residue given the sum of its single-body and pairwise interactions and to account for conformation specificity." How is "relative stability" computed? Is there a reference conformation? Please clarify and move to Methods.

   a. This was a poor wording choice on our part, and we have replaced the "relative stability" that was used throughout the manuscript and supplementary materials with either "relative Gibbs free energy" or just "stability". Rosetta uses a knowledge-based potential to estimate the single and pairwise interaction values within a structure, and so 'relative stability' was used to reflect that the computed per-residue energies are not explicit physics-based energy calculations. No representative conformation was used to to serve as the reference energy.

6) The source of the epitope data is not provided. I did not have access to the Online Methods if there was any.

   a. Tthe Methods subsection Definition of conformation-specific epitopes was revised to clarify the process to how individual residues were labeled for testing purposes, shown below that includes a reference to the direct source of the epitope data.

   b. Lines 414-442: All epitope residues were first identified as any residue that has been annotated as an epitope by the IEDB, Influenza Research Database's Immune epitope search, or the HIV Molecular Immunology Database that is associated with a PDB structure. IEDB searches used the filters 'Positive Assays only', 'Epitope Structure: Discontinuous', 'No T cell assays', 'No MHC ligand assays' and 'Host: Homo sapiens' as of June 1, 2020. Influenza epitope searches used the filters 'Virus Type A', 'Subtype H3 (or H7)', 'Protein HA, Segment 4', 'Experimentally Determined Epitopes', 'AssayType Category and Result B-cell Positive', and 'Host Human' as of June 1, 2020. HIV-1 epitopes include human epitopes as listed in the interactive epitope maps as of June 1, 2020. To determine which each epitope residue's conformation specificity, a residue must have at least one PDB structure of an antibody-antigen complex where it has been annotated as an epitope structure (*i.e.* it has an IEDB/other identification number) and that the PDB of the annotated antibody-antigen complex, when aligned to a protomer, results in zero Å overlap of the antibody with the whole PDB structure. Checking for overlap was performed as follows: i) For each PDB antibody-antigen structure annotated to be associated with a unique epitope ID, the antibody and antigen were created as independent PyMOL objects, for example 'antibody' and 'antigen'. ii) Three PyMOL objects were created for each AxIEM benchmark PDB of identical species to the 'antigen' object, labeled as 'objA', 'objB', 'objC', respectively, for each protomer. iii) The 'antigen' object was first aligned to 'objA'. iv) Next the 'antibody' object was aligned to the 'antigen' object to check for van der Waals overlap with the protomer. The residues annotated within a single epitope ID were considered to associated, or mapped, to that protomer only if zero 'antibody' atoms were within 0.6 Å of any atoms within the PDB containing the protomer of interest. Steps iii and iv were repeated for 'objB' and 'objC'. Note that is was possible for one epitope ID and associated PDB to fail the overlap test

for a single protomer, while a second epitope ID containing similar residues passes the overlap test. If any protomer residue was associated with at least one annotated structural epitope ID, that residue was labeled as an epitope residue, or '1' withing the AxIEM.dataset and other dataset files that store all feature set values, classifier labels, and associated viral fusion protein, PDB ID, and PDB residue ID labels. All annotated epitopes are listed Supplementary Materials.

7) The definition used (distance cut-off criteria) is not specified.

a. We assume that you are referring to the distance cut-off criteria used to determine the Neighbor Sum metrics, not the CP variation or NV metrics, which include an explanation of the CP and NV cut-off criteria in the Methods section. We have included the reasoning behind using the upper and lower boundary cut-off used to compute the NS metrics in greater detail in the first and second Results section.

b. Lines 156-160: To avoid making assumptions about what distance away neighboring residues contribute to the antigenicity of a residue, we used an upper boundary cutoff of incremental distances of 8 Å, or half of the radius of an average antibody binding footprint, up to 64 Å to determine which optimal neighborhood volume to use to compute NS. We used a lower boundary cutoff of 4.0 Å to limit the weighted contribution of 1 to only the residue itself, not its neighboring residues.

c. Lines 212-213: we found that the mean AUC scores for all methods peaked using an *NS* upper boundary radius of 24 Å

8) My impression is that the epitope data is incomplete. For instance, for SARS-CoV-2 spike protein, the test set (https://github.com/mfsfischer/AxIEM/blob/main/testing_datasets/SARS2_epi.csv) contains 525 positive labels for 21123 negative ones. Given that there are 8 trimers here, this leaves 525/8*3 ~ 22 unique epitope residues per chain. I am certain that they are many more epitope residues, based on available crystal structures or a quick iedb search. Same for SARS-CoV-1 (15 epitopes). RSV and HIV datasets looked reasonable.

a. The epitope data we used initially included all deposited IEDB IDs as of June 1, 2020. Apart from the SARS-related S proteins, the number of deposited epitopes within the IEDB has remained the same, and all IEDB IDs were used in our study. Please note that for many IEDB IDs and the associated PDBs , the majority of structural epitope data publicly available is highly redundant and includes the exact same epitope residues as defined in Response 6b. Additionally, we do not assume each trimer is symmetric, and therefore it is not reasonable to expect that all chains should contain the same number of epitope residues.

b. Regarding the GitHub documentation and source code, a substantial effort during revisions was put into restructuring the scripts and improving the documentation during revisions so that the data produced by this benchmark is clear and 100% reproducible by another individual. More importantly we provide a simple script that outputs the residue-wise probabilities.

9) The definition of conformation-specific epitope L374 is unclear. What quantitative criteria are used to define structural similarity and/or antibody overlap with the antigen? Are the results shown for conformation-specific epitopes only, or for all epitopes?

a. Please refer to Response 6b for the updated definition of a conformation-dependent epitopes – L374 was defined this way.
b. Results are shown for conformation-dependent epitopes only. This manuscript does not attempt to predict linear peptide epitopes.

10) There are hundreds of structures of fusion protein structures available on the pdb. What was the rationale for selecting these seven structures?

a. Seven class I fusion protein ensembles were used in this benchmark, represented by 34 PDB structures. Yes, there are hundreds of fusion protein structures, but the method proposed here requires multiple conformations of full-length trimeric class I fusion proteins – which excludes many fusion protein structures as they are monomeric protomers or subunits which cannot be used to accurately estimate relative surface exposure. Additionally, the seven protein ensembles used in this manuscript represent all class I fusion proteins that have been determined with the structural diversity requirements our method requires. We understand that this structural requirement poses a major limitation to the broad usefulness of the proposed method. However, with computational structure prediction methods like AlphaFold 2.0, which can be readily be used through a server, it is feasible to determine probable conformational ensembles of fusion proteins that have not yet been determined in multiple conformations, which we bring up in the Discussion.

11) The manuscript features multiple 40/50+ word sentences that are very hard to parse/understand.

a. We have made a concerted effort to avoid these types of overly complicated sentences.
b. L306-309 This sentence and related paragraph were removed for clarity.
c. L49: typo "techniques, ," – comma removed
d. L51-55: sentence unclear. – paragraph was revised for clarity (now lines 47-57) To consider how protein flexibility might influence antigenicity, viral fusion proteins must have been determined in conformations that correspond with multiple stages of attachment and/or fusion – and have been extensively subjected to B-cell epitope mapping techniques. Despite advances in cryptic epitope determination, the available data is limited to a subset of class I fusion proteins that meet the above criteria. This poses a challenge to computational epitope mapping in generating an informative model that avoids overfitting. Here, we discuss a limited set of descriptive features, that when used in a variety of low complexity classifier models, matches or outperforms other publicly available B-cell epitope prediction methods in out-of-sample tests. From the models we tested, we use the linear regression model to highlight structural insights of epitopes and to demonstrate how this model may provide a novel approach to assess structural changes of antigenicity between viral fusion protein homologues.
e. L65-67: sentence unclear – replaced with paragraph (lines 64-75) B-cell epitopes are defined as a spatially clustered set of antigen residues with a surface area of 600 $Å^2$ to 1,000 $Å^2$ that interact with an average antibody binding footprint of 800 $Å^2$. (2) Epitope mapping of viral fusion proteins, however, indicates that multiple epitopes may overlap in a single domain or interface region, known as a site of vulnerability, but differ in binding site area and the combination of antigen residues that comprise a specific antigen-antibody interaction. (3–8) The major challenge of B-cell epitope mapping

prediction is to identify residues that are most likely to form a direct contact with at least one neutralizing or protective antibody without assuming the conformation of the paratope. In addition, especially for emerging or under characterized antigens, the goal of computational epitope mapping is to not only have the sensitivity to detect previously determined sites of vulnerability, but also to provide reasonable predictions of novel sites of vulnerability.

f. L161-164: sentence unclear. English; "despite that" – Sentence was removed.

g. L203-207: The sentence and subsequent discussion cannot be understood. "the closed conformation … were labelled to be antigenic, while only the 2 or 3-RBD up … were labelled to be antigenic". – Sentence was replaced with: SARS-CoV and SARS-CoV-2 S proteins provide a unique challenge for structural epitope mapping because multiple conformations of each trimeric S protein have been determined with various orientations of the receptor binding domain (RBD), with each conformation associated with unique mechanisms of neutralization. (67–69) Structurally determined conformations includes the 'closed', '1-RBD open', and '2-RBD open' conformations for both S proteins, (70–74) and the '3-open'/'fully open' conformation for SARS-CoV-2 S protein. (75) The majority of available structures of Ab-S protein interactions, however, exist as fragment Ab and RBD complexes and are not annotated as specific to any specific conformation. (9) To map the fragment Ab-RBD epitopes to a reference S protein conformation requires superimposition of the RBD from the fragment Ab-RBD interaction to each of the RBDs within each conformation to determine if the epitope is accessible, both in terms of epitope surface accessibility and potential Ab clashes with other protomers. Given the criteria used by this study to map epitopes to each conformation, most fragment Ab-S protein complex epitopes were exclusively mapped to the closed conformation for both SARS-CoV and SARS-Cov-2 S proteins, which were also used to define the expected classification label of whether a residue should be predicted to be an epitope or not.

h. L239-245. I could not understand neither the definition of FP_{FP <-> FN} and FN_{FP <-> FN}, nor the point that the authors were trying to make here. This argument and associated table were removed as we agree this was an unclear argument.

i. L269 typo – The extra period was removed.

j. L295: elicit – Replaced illicit with elicit.

12) Eqn 3 L338: The left-hand term of the equation (NV_i) is a scalar, whereas the right-hand is a vector. A norm operation is missing? Yes it was. Thank you for catching this, and the norm operator was added to the right-hand term of Eq. 3.

13) Eqn 4 L 352: the indexing of the equation is not consistent with the text description. The inner sum should be over the conformation index, the outer sum over the neighbor index j. One running index for conformations k=1..n should be introduced.

14) L354: Terminology; (Av(CP(ji)) – CP(ji)_n)^2 is not the variance of the CP value but the square deviation (the variance corresponds to average of the square deviation). The square root operator was removed since we changed the calculation of the CP RMSD to CP variation and the equation now reflects that we use the variance.

15) The authors should clearly state what was used for training and the withheld test sets for their results. It is not clear whether the AxIEM results shown in Table 1, Figure 2, and Figure 3 are from re-training of the model separately with distinct antigens and epitopes to apply to the test cases, or if there is a possible overlap between the training and the cases being shown in the results. One concern is that SARS-CoV and SARS-CoV-2 antigens could be present in both the training and test sets, and they have similar sequences and structures, as well as overlapping known epitopes in the RBD, etc. Additionally, there are two influenza HA antigens (HA stem from H3, and full H7 HA), which could also represent a possible redundancy in epitopes and structures, and it is not clear if they were separated during the training and test regimen. Details regarding the training and testing would be very helpful for readers to gauge the overall success of AxIEM and its comparison with Discotope and Ellipro, as nonredundancy between the train/test sets, or ideally the use of a withheld set, would be critical in gauging the predictive success of AxIEM on viral antigens not seen during its training.

   a. We appreciate that you brought up this concern. We included the following text in the Results section to better clarify how the training and testing sets were prepared: Constructing each leave-out training dataset involved removing all feature data originating from any structures representing one of the class I fusion proteins from the entire dataset. The removed features were then retained as the corresponding leave-out testing set. When either influenza H3 and H7 hemagglutinin or Severe Acute Respiratory Syndrome (SARS)-related Spike (S) proteins, SARS-CoV S and SARS-CoV-2 S, were used for leave-out testing, the related hemagglutinin or S protein was removed from the training dataset and not included within the testing dataset to ensure non redundancy between the training and testing data for each leave-out test.
   b. We updated the figure comparing AxIEM with Discotope and Ellipro using a more straightforward comparison that indicates the average AUC performance for all leave-out tests using either AxIEM, Discotope, or Ellipro. The AxIEM AUC and average AUC values that we report are calculated using out-of-sample test data. No retraining was performed.

16) The Methods section on “Definition of conformation-specific epitopes” requires more explanation and clarity. Regarding the experimentally validated epitopes used in this study, the authors note: “…database identifications have listed in Online Methods”. However, this information does not appear to be in the supplemental materials. Please provide more information where this information is located, or include it if it is not present.

   a. Please see below for the updated Methods section explaining the

Definition of conformation-specific epitopes

All epitope residues were first identified as any residue that has been annotated as an epitope by the IEDB, Influenza Research Database’s Immune epitope search, or the HIV Molecular Immunology Database that is associated with a PDB structure. IEDB searches used the filters ‘Positive Assays only’, ‘Epitope Structure: Discontinuous’, ‘No T cell assays’, ‘No MHC ligand assays’ and ‘Host: Homo sapiens’ as of June 1, 2020. Influenza epitope searches used the filters ‘Virus Type A’, ‘Subtype H3 (or H7)', ‘Protein HA, Segment 4’, ‘Experimentally Determined Epitopes’, ‘AssayType Category and Result B-cell

Positive', and 'Host Human' as of June 1, 2020. HIV-1 epitopes include human epitopes as listed in the interactive epitope maps as of June 1, 2020.

To determine which each epitope residue's conformation specificity, a residue must have at least one PDB structure of an antibody-antigen complex where it has been annotated as an epitope structure (*i.e.* it has an IEDB/other identification number) and that the PDB associated with the epitope, when aligned to a protomer, results in zero Å overlap of the antibody with the whole PDB structure. Checking for overlap was performed as follows: i) For each PDB antibody-antigen structure annotated to be associated with a unique epitope ID, the antibody and antigen were created as independent PyMOL objects, for example 'antibody' and 'antigen'. ii) Three PyMOL objects were created for each AxIEM benchmark PDB of identical species to the 'antigen' object, labeled as 'objA', 'objB', 'objC', respectively, for each protomer. iii) The 'antigen' object was first aligned to 'objA'. iv) Next the 'antibody' object was aligned to the 'antigen' object to check for van der Waals overlap with the protomer. The residues annotated within a single epitope ID were considered to associated, or mapped, to that protomer only if zero 'antibody' atoms were within 0.6 Å of any atoms within the PDB containing the protomer of interest. Steps iii and iv were repeated for 'objB' and 'objC'. Note that it was possible for one epitope ID and associated PDB to fail the overlap test for a single protomer, while a second epitope ID containing similar residues passes the overlap test. If any protomer residue was associated with at least one annotated structural epitope ID, that residue was labeled as an epitope residue, or '1' withing the AxIEM.dataset and other dataset files that store all feature set values, classifier labels, and associated viral fusion protein, PDB ID, and PDB residue ID labels. **All annotated epitopes are listed in Supplementary Materials.**

17) This sentence from that Methods section (lines 374-378) requires clarification: "The definition of an epitope was further refined as any residue identified as a discontinuous epitope that also retained a structural similarity to the conformation of the antigen experimentally determined to interact with a specific antibody and that the binding orientation of the antibody did not occupy the same space, or intersect, with any part of the full-length viral protein in that conformation when aligned using PyMOL."
   a. Please refer to the previous response 16a that includes clarification on previous lines 374-378.

18) It is not clear what "specific antibody" the authors used for these comparisons; the set of these should be provided. Also, it is not clear what metric was used to define structural similarity (e.g. RMSD, within some cutoff distance).
   a. We included the IEDB accession IDs within the GitHub repository for the initial submission, but we have moved the information containing the IDs to the supplementary materials along with instructions to access the IDs, which provide all PDB structures associated with an individual ID. We do not believe it is necessary to reannotate an already existing database regarding antibody identities.
   b. We expanded the section describing the criteria used to define structural similarity: The structures were selected based on the following criteria so that each protein ensemble included: i) at least two multimeric conformations of greater than 2.00 Å root mean square deviation (RMSD), ii) no two conformations of less than 1.00 Å RMSD, iii) every PDB had a resolution of below 4.5 Å, and iv) each conformation, such as an 'open' or 'closed' conformation, were equally represented within a protein ensemble (26–46).

The first criterion was imposed to maximize the structural diversity of each fusion protein ensemble, given that each protein includes at least one side chain contact rearrangement of over 10 Å between the precursor or prefusion conformation and the postfusion conformation. And in Methods: Curation of viral fusion protein conformations began with a Protein Data Bank query of viral fusion proteins in their trimeric state that were identical in virus family and strain or serotype. All PDB structures that shared ≥ 95% amino acid sequence identify were downloaded from the Protein Data Bank and were superimposed in a pairwise fashion using the PyMOL align command with number of cycles set to zero. For PDB structures sharing less than 1.0 Å RMSD, the structure with the least number of missing densities was selected to represent a single fusion protein conformation. The final set of selected fusion protein ensembles included ensembles with at least a single 2.0 Å pairwise RMSD between two conformations, and no two conformations shared less than 1.0 Å RMSD. Additionally, for ensembles such as SARS-CoV-2 that have multiple, distinct conformations the number of distinct conformations were equally balanced in conformation representation.

19) As the authors appear based on this description to utilize their own criteria to define epitopes, so they should list the final set of epitope residues for each antigen (used in their training and testing analysis) in their supplemental information.

   a. We added this information to the Supplementary Materials.

20) The Introduction section needs more references to provide readers with context regarding several of the statements being made by the authors. For example:

   a. Lines 68-70: A citation should be added for the IEDB where it is noted. – Citation was added.
   b. Lines 82-83: "Compared to other proteins with antigenic determinants within a viral quasispecies, fusion proteins are more frequently targeted by broadly neutralizing antibodies…". One or more references should be added in support of this statement.
   c. Lines 84-85 ("…so-called 'reverse vaccinology'."). Please provide at least one reference - regarding reverse vaccinology. One possibility is: Rappuoli et al. J Exp Med (2016) 213 (4): 469–481. – Five references were included to support lines 82-85, or current lines 91-94.
   d. Lines 88-90: "Recent advances in experimental design and cryogenic electron microscopy (cryo-EM) allow discovery of cryptic epitopes in 'alternative' conformations of viral fusion proteins." A reference or multiple references providing examples for this would be very helpful here. – Six references were added of recent EM structures of cryptic epitopes

21) As noted by the authors (Line 410), the code and data for running this protocol are available on Github, which is great. However, the Github repository does not appear to contain any README file or information for interested readers on how to run the protocol. The authors should provide a README or documentation for this repository, in accordance with standard practices, to enable readers to understand and run the algorithm being described in this manuscript.

   a. A README was added to the repository with a short explanation of how to use AxIEM. A detailed protocol capture is included in the repository. Additionally, as mentioned in a previous response, the repository was restructured so that it is clear what data and

scripts are being used.
